# Multiple anomalous left pulmonary venous connections detected with transthoracic echocardiography

**DOI:** 10.1186/1749-8090-8-130

**Published:** 2013-05-17

**Authors:** Tzu-Lin Wang, Huei-Fong Hung, Chang- Chyi Lin, Ming-Chon Hsiung, Jeng Wei

**Affiliations:** 1 Shin Kong Wu Ho-Su Memorial Hospital, Taipei, Taiwan; 2 Heart Center, Cheng Hsin General Hospital, No. 45, Cheng Hsin St, Pai-Tou, Taipei, Taiwan

**Keywords:** Innominate vein, Partial anomalous pulmonary venous connection, Transthoracic echocardiography

## Abstract

Partial anomalous pulmonary venous connection is a rare congenital anomaly in which one or more pulmonary veins are connected to the venous circulation. The condition is frequently misdiagnosed, and usually identified by transesophageal echocardiography or invasive cardiac catheterization. We present the case of a 26-year-old female with new onset dyspnea on exertion who was diagnosed with the left superior and inferior pulmonary veins draining into the innominate vein via a vertical vein by two and three-dimensional transthoracic echocardiography and multidetector computed tomographic angiography.

## Background

Partial anomalous pulmonary venous connection (PAPVC) is a rare congenital anomaly in which pulmonary veins carry blood from the lungs to the right side of the heart. The condition has a prevalence of 0.4-0.7%, it is frequently diagnosed as an incidental finding, and right side PAPVC is more common than left [1,2] A single PAPVC is found in 53% of cases, and two in 42%; more numerous anomalous connections are very rare [2] Herein we report an adult patient in which both the left superior and inferior pulmonary veins (LSPV and LIPV) drained into the innominate vein that was diagnosed by non-invasive modalities: transthoracic echocardiography (TTE) and multidetector computed tomographic (CT) angiography.

## Case presentation

**Figure 1:**
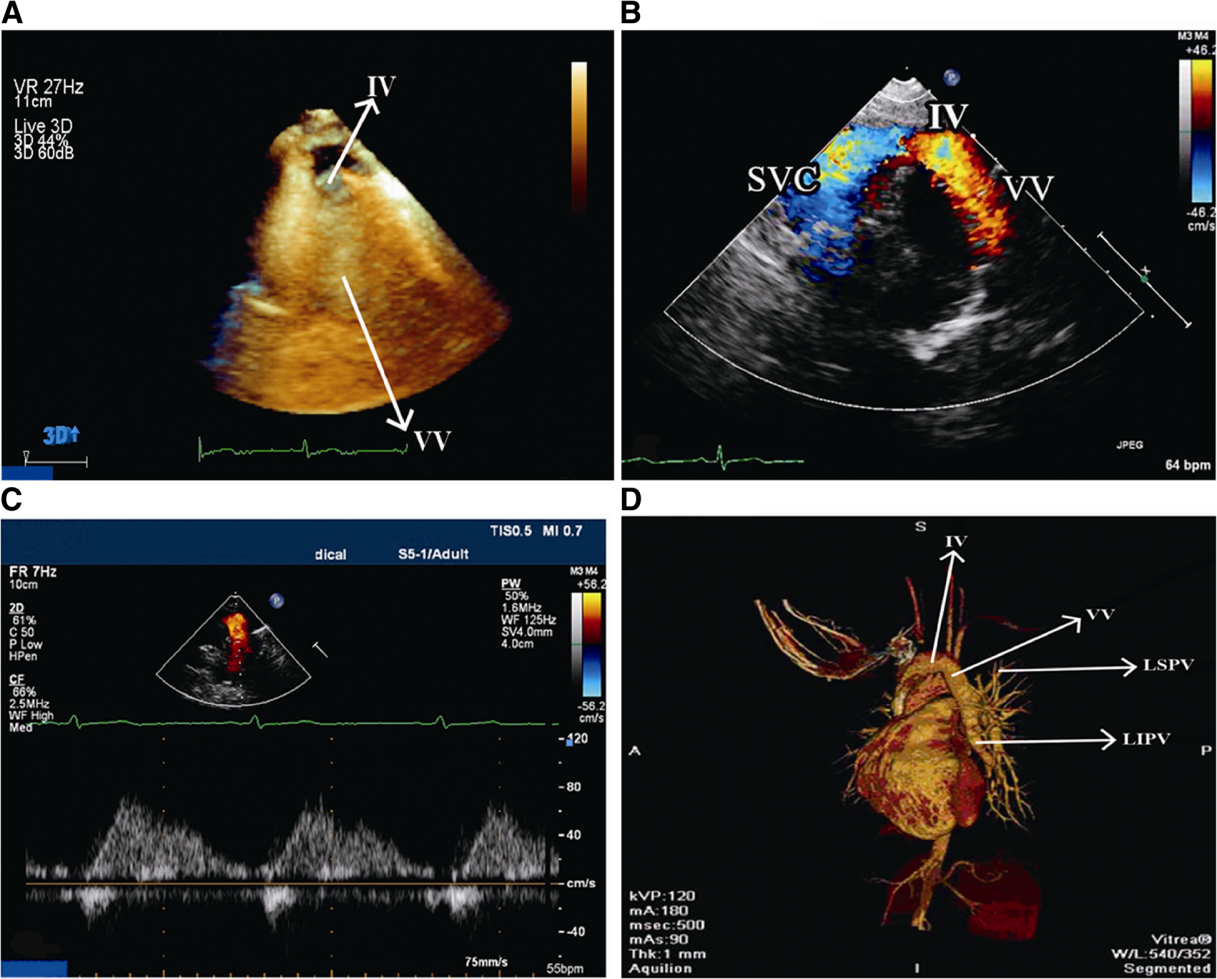
**Four images for detecting partial anomalous pulmonary venous connection (PAPVC).** (**A**) Three dimensional transthoracic echocardiography showed PAPVC. (**B**) The suprasternal short-axis view with color Doppler revealed left pulmonary veins connecting with the innominate vein via a vertical vein. (**C**) Upward blood flow in the vertical vein was confirmed by pulsed Doppler imaging. (**D**) Multidetector computed tomography angiography was performed to confirm the structure of the anomalous venous system. IV: innominate vein; SVC: superior vena cava; VV: vertical vein.

**Figure 2:**
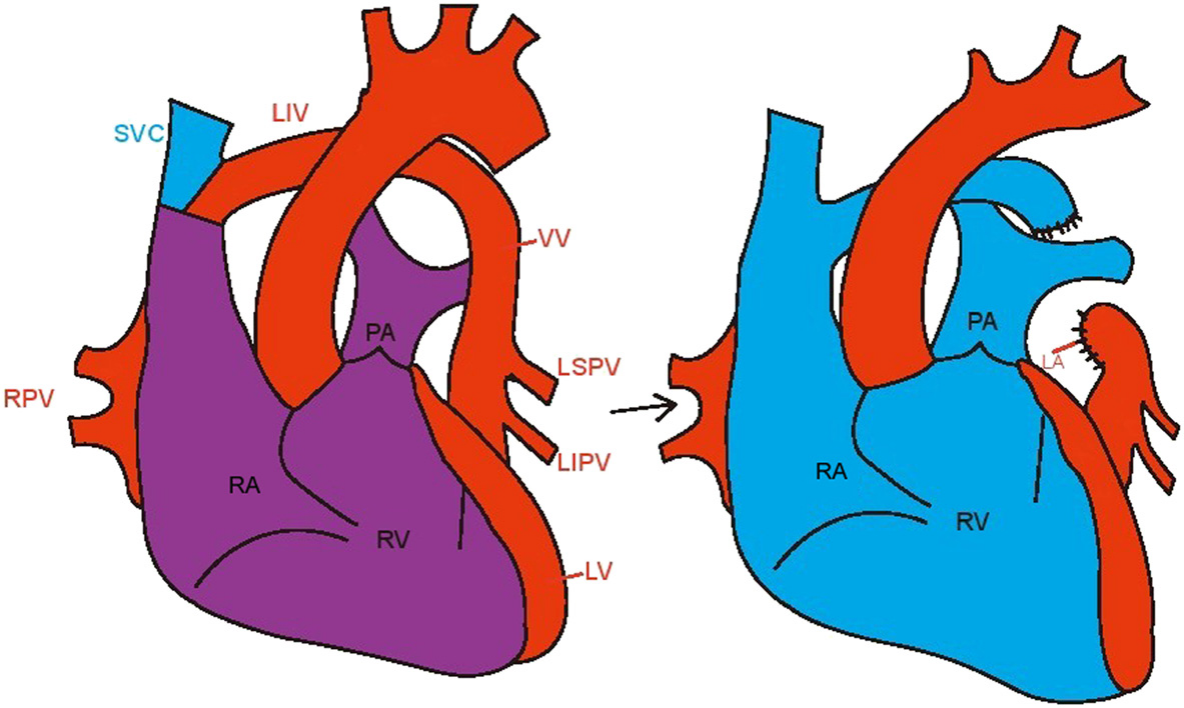
**Surgical correction of the partial anomalous pulmonary venous connection (PAPVC).** The diagram of PAPVC (left). The distal end of the vertical vein was anastomosed to the left atrial appendage (right). SVC: superior vena cava; LIV: left innominate vein; VV: vertical vein; LSPV: Left superior pulmonary vein; LIPV: Left inferior pulmonary vein; RPV: Right pulmonary vein;PA: pulmonary artery; RA: Right atrium; RV: Right ventricle; LV: left ventricle.

A 26-year-old female with no history of cardiovascular disease was referred to our hospital for mild dyspnea during exertion over a period of 3 months. She had been treated at another institution with propranolol and alprazolam for a presumptive diagnosis of mitral valve prolapse without relief. On admission her blood pressure was 110/64 mm Hg, heart rate was 86 beats/min, and respiratory rate was 18 breaths/min. Physical examination was unremarkable. A 12-lead electrocardiogram showed normal sinus rhythm with incomplete right bundle branch block. Chest radiography was normal. TTE showed normal left and right ventricular function, but the right ventricle was mildly dilated. Right ventricular systolic pressure was 35 mm Hg with moderate tricuspid regurgitation. There was no evidence of an interatrial septal defect, although the calculated Qp/Qs was 1.85. Suprasternal short-axis view revealed direct drainage of both the LSPV and LIPV into the innominate vein (IV) via a vertical vein (VV) (Figure 1A). Color Doppler imaging in the same plane also showed red flow in IV and VV (directed toward the transducer) and blue flow in superior vena cava (directed away from the transducer) (Figure 1B) Pulse Doppler showed a venous flow pattern upward in the vertical vein (Figure 1C). Multidetector CT angiography was performed to to confirm the echocardiographic findings of anomalous circulation (Figure 1D). She underwent surgical correction during which the distal end of the vertical vein was anastomosed to the left atrial appendage (Figure 2). Postoperative echocardiography showed the Qp/Qs below 1.0, and her symptoms had resolved after 6-months follow-up.

### Discussion

PAPVC is an important diagnosis, but is often overlooked in clinical practice. Early presentations resemble those of patients with an atrial septal defect (ASD), exercise intolerance, or atrial arrhythmia, and later in the course of the disease, right heart failure and pulmonary hypertension. Symptoms relate to the magnitude of left-to-right shunting [3]. The most common associated anomaly is an ASD [2,4]. The reported incidence of sinus venosus ASD in patients with PAPVC ranges from 49% to 85%, and that of secundum ASD ranges from 10% to 33% [2,4-6]. Though rare, cases of PAPVC associated with valvular heart disease have been reported [7]. It is important to understand whether the anomalous pulmonary connection coexisted with or without an interatrial shunt before planning the surgical correction. The reported sites of PAPVC include the right superior vena cava, left subclavian vein, left innominate vein, azygous vein, portal vein, and coronary sinus [2].

Invasive cardiac catheterization and transesophageal echocardiography are often used to diagnosis pulmonary vein return anomalies; however, the procedure is performed under sedation and is associated with inherent risks. In this case, TTE efficiently detected the anomaly in suprasternal short-axis view. TTE can identify right atrial and ventricular enlargement, increased pulmonary pressure, and interventricular septal flattening as a result of increased right ventricular pressure [4]. The suprasternal and subcostal views are very important in diagnosing PAPVC. For example, subcostal view can identify pulmonary venous connections and septal abnormalities and the suprasternal short-axis view offer excellent imaging of all four pulmonary veins and can identify abnormal structures such as a vertical vein with upward blood flow [4]. Nevertheless, it is more challenging to perform these views of echocardiography in adults than in children because of grown body size and body composition. Several conditions, for example, obesity, chronic obstructive pulmonary disease, and chest wall deformities can also reduce the accuracy of TTE. Patients with unexplained right heart dilatation should prompt the sonographer to search extensively for evidence of a left-to-right shunt, most likely across an ASD, and/or for the presence of PAPVC. Additionally, in this patient while Qp:Qs > 1 there was no evidence of flow across the interatrial septum; thus, the findings were suspicious for a PAPVC and then an comprehensive searching for pulmonary venous connections should be performed.

As a complement to TTE, color Doppler and three-dimensional echocardiography are very useful for identifying the presence of these abnormalities [4]. In addition, CT can provide optimal visualization of the complete thoracic vessels by reconstruction, as well as cardiac magnetic resonance imaging [8].

Surgical correction is necessary in cases where Qp/Qs is > 1.5:1 and the right ventricle is enlarged [5]. Reports of repair in adults have indicated good outcomes [9]. The degree of left-to-right shunting determines the type of management [3]. Patients with anomalous drainage of more than 50% of the pulmonary blood flow should have surgical repair to prevent following complications. For patients with more than one anomalous pulmonary venous connection but less than 50% anomalous drainage of pulmonary blood flow, the degree of left-to-right shunting may increase during adulthood because of changes in left ventricular compliance. These patients should remain under periodic review and surgery is indicated while symptoms such as dyspnea on exertion develop. Asymptomatic cases such as isolated PAPVC with low level of Qp/Qs can be managed conservatively with annual follow-up [3].

## Conclusions

In conclusion, PAPVC involving the LSPV and LIPV can be easily diagnosed by noninvasive TTE accompanied with CT imaging in an adult.

### Summary

Partial anomalous pulmonary venous connection is a rare and frequently misdiagnosed congenital anomaly in which pulmonary veins are connected to the venous circulation. We present the case of a 26-year-old female with new onset dyspnea diagnosed with the left superior and inferior pulmonary veins draining into the innominate vein by two and three-dimensional transthoracic echocardiography and multidetector computed tomographic angiography.

## Consent

Written informed consent was obtained from the patient for publication of this Case report and any accompanying images. A copy of the written consent is available for review by the Editor-in-Chief of this journal.

## Abbreviations

PAPVC: Partial anomalous pulmonary venous connection; LIPV: Left inferior pulmonary vein; LSPV: Left superior pulmonary vein; TTE: Transthoracic echocardiography; CT: Computed tomography; IV: Innominate vein; VV: Vertical vein; ASD: Atrial septal defect.

## Competing interests

The authors declare that they have no competing interests.

## Authors’ contributions

TLW drafted the manuscript and performed literature search. HFH and CCL supervised and reviewed the manuscript. MCH performed echocardiography, analyzed and interpreted the imaging results and supervised the manuscript. JW performed the surgery and coordinated the study. All authors read and approved the final manuscript.
